# Role of Temperature-Dependent Interfacial Tension on Shear Wave Velocity for Energy Geosystems

**DOI:** 10.3390/s23218709

**Published:** 2023-10-25

**Authors:** Junghee Park, Jongchan Kim

**Affiliations:** 1 Department of Civil and Environmental Engineering, Incheon National University, Incheon 22012, Republic of Korea; junghee.park@inu.ac.kr; 2 Department of Civil and Environmental Engineering, University of California at Berkeley, Berkeley, CA 94720, USA

**Keywords:** degree of saturation, repetitive load deformation, shear wave velocity, interfacial tension, temperature

## Abstract

Interfacial tension varies with temperature. This paper investigates the effects of temperature-dependent interfacial tension on shear wave velocity. We designed a nylon cell equipped with bender elements in a cross-hole configuration to measure the shear wave velocity of nine sand–silt mixtures with different degrees of saturation (*S* = 0%, 2.5%, 5%, 10%, and 100%). All specimens were subjected to a temperature change from 10 °C to 1 °C. The results demonstrate that shear wave velocity tends to be very sensitive to changes in temperature at a low degree of saturation. Particle-scale analyses overlapped with the experimental results and captured the critical role of temperature-dependent interfacial tension in small-strain skeletal stiffness. In fact, the temperature should be considered during laboratory and field shear modulus measurements of the long-term performance of energy geosystems subjected to thermally induced repetitive loads.

## 1. Introduction

In geotechnical engineering, the elastic modulus is an important design parameter that represents the load deformation characteristics of the soil [1]. Under dynamic load conditions, the maximum shear modulus (*G_max_*) is a useful soil property that is relevant to various dynamic designs, including the foundation, pile installation, transportation infrastructure, and analysis of earthquake-induced soil liquefaction [2]. The maximum shear modulus (*G_max_*) at small strains can be calculated from the shear wave velocity measured using the bender element. Previous investigations have used shear waves to evaluate the shear modulus for sand–rubber mixtures and the elastic modulus for subgrade soil with changes in water content [1,3]. The shear modulus is primarily affected by the soil type (i.e., particle size and distribution), state of stress (i.e., effective stress), and degree of water saturation [4,5]. In particular, the degree of saturation appears to be critical in the determination of the *G_max_* obtained via shear wave velocity measurements [6].

In general, shear wave velocity increases with effective stress. More specifically, shear wave velocity follows the power function of effective stress, and the effective stress of unsaturated soils can vary depending on the degree of saturation and matric suction between the soil particles. Matric suction is proportional to the interfacial tension, and this capillary-driven force is a function of the interfacial tension, which varies with temperature. Therefore, it is clear that temperature-dependent interfacial tension is relevant in the maximum shear modulus. However, the role of temperature-dependent interfacial tension in small-strain stiffness remains unclear.

Changes in shear wave velocity were investigated for dry (degree of water saturation *S* = 0%), unsaturated (0 < *S* < 100%), and fully water-saturated (*S* = 100%) specimens subjected to a temperature change from 10 °C to 1 °C. This paper describes the design of a nylon cell equipped with a set of bender elements in a cross-hole configuration to measure the shear wave velocity for nine sand–silt mixtures with different degrees of saturation (*S* = 0%, 2.5%, 5%, 10%, and 100%). This manuscript starts with the theoretical background of interfacial tension acting on soil particles, followed by the characteristics of the soil mixtures used in the experiment, the specimen preparation method with different degrees of saturation, and the characteristics of the experimental cell design for shear wave velocity measurement. In addition, particle-scale analyses relevant to capillary pressure prediction in pores are compared with the experimental results.

## 2. Temperature-Dependent Interfacial Tension in Soils

**Figure 1:**
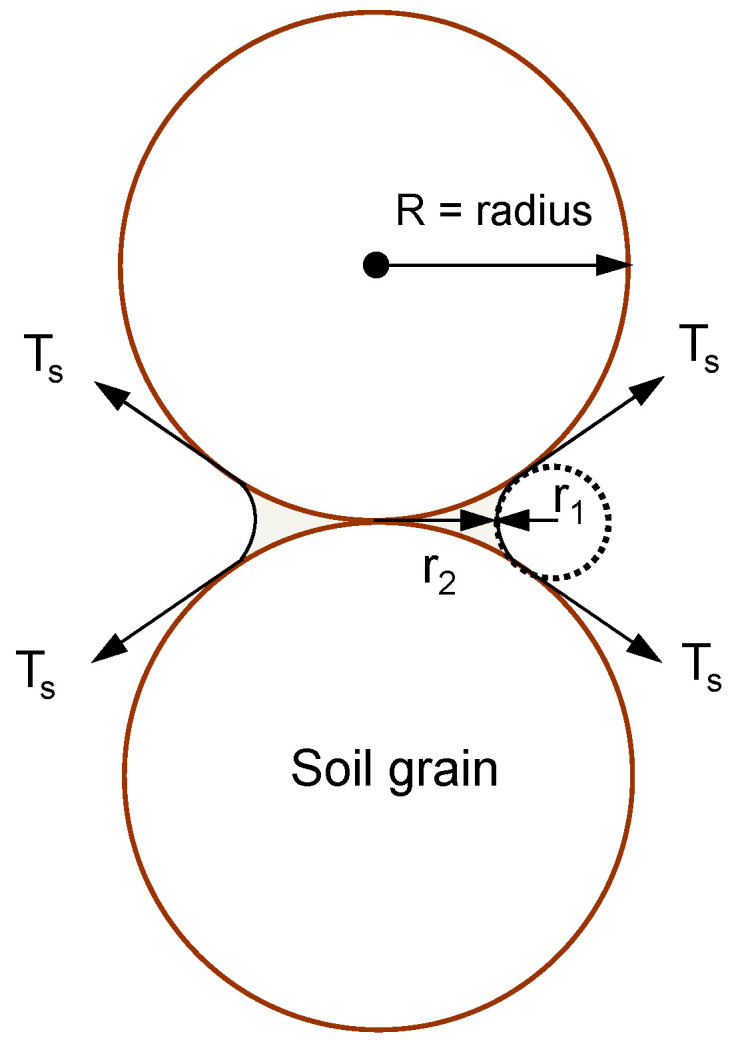
Interfacial tension acting on soil grains (*T_s_*: interfacial tension, *R*: radius of soil grain, *r*_1_: inner meniscus radius of curvature, *r*_2_: outer meniscus radius of curvature).

Interfacial tension builds up at the point of grain contact under unsaturated conditions. Assuming that soil particles are spherical (Figure 1), the contact point of the two particles is surrounded by pore water, and the interfacial tension acts along the tangent of each sphere. A compressive force is generated against this interfacial tension, and this phenomenon increases the effective stress between soil particles. If there is adequate moisture in soils, there is a force that adheres the soil particles to each other due to interfacial tension; however, this force diminishes in a completely dry or saturated state (i.e., apparent cohesion), and it is also mainly affected by the degree of water saturation. Previous research has identified effective stress under unsaturated conditions using matric suction, which increases the effective stress between soil particles [7].

**Figure 2:**
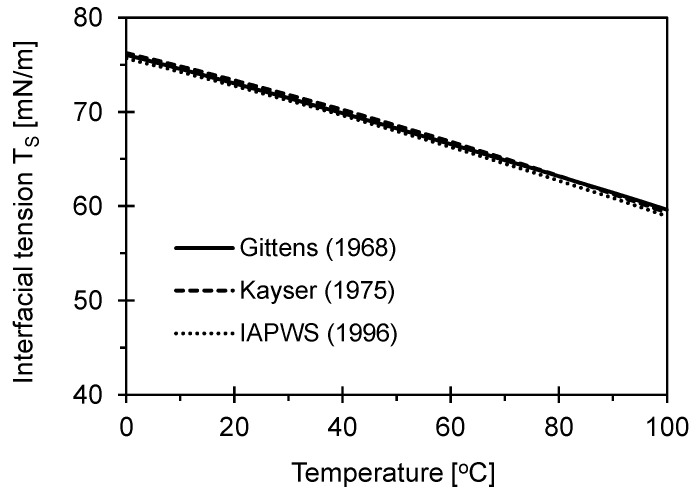
Changes in interfacial tension due to temperature changes.

Interfacial tension varies with the degree of saturation or water content, and the magnitude of the force changes with temperature [8]. As shown in Figure 2, the interfacial tension increases as the temperature decreases, ranging from the boiling point (100 °C) to the freezing point (0 °C) of water. The relationship between temperature and interfacial tension can be expressed as a polynomial function of temperature [8,9,10]. Clearly, the interfacial tension decreases as the temperature increases. There have been many previous investigations into interfacial tension and temperature, yet the trends are similar to each other.

## 3. Materials and Methods

This section briefly reports the materials used for specimen preparation and the methods adopted in the experimental research.

### 3.1. Tested Materials

We used standard Jumunjin sand (grain particle diameter *D* = 0.30∼0.60 mm) with a grain size between the No. 30 and No. 50 sieves and crushed limestone (silt materials in this test) that passed through the No. 200 sieve. The mean grain-particle diameter was *D_50_* = 0.45 mm. Sand and silt were uniformly mixed, so the ratio between the silt weight and sand weight (% of silt = *W_silt_*/*W_sand_* × 100%) was 10% in all cases. The specific gravity of the sand–silt mixture was 2.57 [11], the maximum void ratio was *e_max_* = 0.74 [12], and the minimum void ratio was *e_min_* = 0.47 [13]. Table 1 summarizes the material properties of the sand and sand–silt mixtures.

### 3.2. Specimen Preparation

The specimens were prepared with the relative density of the uniformly mixed sand–silt mixtures equal to *D_r_* = 70% (i.e., dry unit weight *γ_d_* = 16.3 kN/m^3^). Then, nine different sand–silt mixtures were prepared with various degrees of saturation (*S* = 0%, 2.5%, 5%, 10%, and 100%) using the following volumetric–gravimetric relationship:

(1) $$S=\frac{\omega \cdot G_{s}}{e_{\left(D_{r}=70\%\right)}}$$

where *S* is the degree of saturation, *ω* is the water content, *G_s_* is the specific gravity of the sand–silt mixture, and *e*_(*Dr*=70%)_ is the void ratio corresponding to a relative density of *D_r_* = 70%. The tamping method was used for a sand–silt mixture placed in four layers of a cell to reach the target relative density.

### 3.3. Shear Wave Measurement Cell

**Figure 3:**
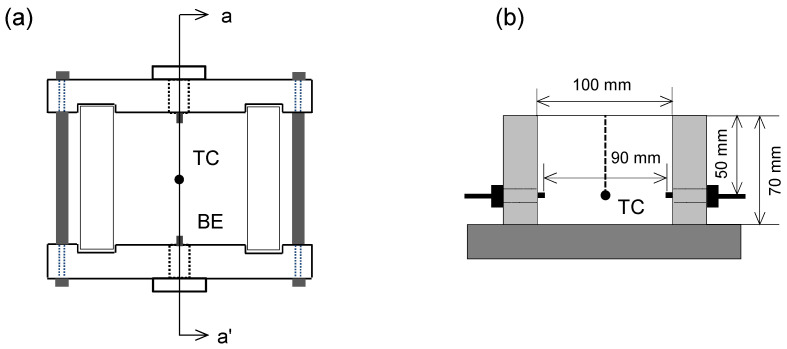
Shear wave measurement cell: (**a**) top view and (**b**) cross-section side view along a–a′ line plotted in (**a**).

The instrumented shear wave measurement cell is used, as depicted in Figure 3. The cell is made of nylon and has a square width and length of 100 mm on the inside and a height of 70 mm. The cell was designed to house a pair of bender elements (BE) for a shear wave transducer in a cross-hole configuration. Thus, the shear wave signatures propagate in the horizontal direction of the soil specimens and are captured at the received bender element. The bender element used as a shear wave transducer shows the successful coupling effect between the soil particles and the transducer [2]. Furthermore, a thermocouple (TC) is installed at the center of the cell to monitor the temperature fluctuations of the specimens (*K*-type, thermometer CENTER-309).

### 3.4. Test Procedure

**Figure 4:**
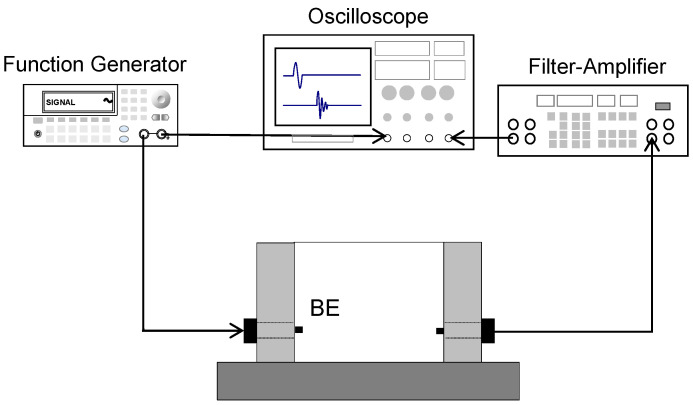
Shear wave measurement system. A function generator generates the shear wave signatures that propagate through the tested specimens and are captured via the received BE sensors. Then, the propagated signatures are filtered and amplified using a filter amplifier and shown in the oscilloscope.

The shear wave was measured for nine specimens with different degrees of saturation over temperature changes from 10 °C to 1 °C. An environmental chamber was used to control the temperature of the specimen. The ambient temperature was set to −20 °C. The cell was insulated to prevent the side of the cell from being frozen by the cooling air. No external vertical stress was applied to the specimens because the main objective of this research was to evaluate temperature effects on shear wave velocity. Plastic wrap was placed on the top surface of the instrumented cell to prevent water evaporation during cooling. The shear wave measurement system included the function generator, filter and amplifier, and oscilloscope (Figure 4). The selection of a parallel type of bender element to generate and detect shear waves is considered to be under the influence of crosstalk [2]. The dimensions of the bender elements were 10 × 7 × 0.7 (length × width × thickness in mm), with a 5 mm cantilever length. The function generator formed step input signals with a 10 input voltage (Keysight 33210A, Santa Rosa, CA, USA), and elastic shear waves were transformed by the bender element and then propagated through the soils. The propagated shear wave signatures were captured by the received sensors and transmitted to the filter amplifier to filter and amplify the signatures. The filter amplifier (Krohn-Hite 3364, Brockton, MA, USA) used 500 Hz and 200 kHz for high- and low-pass filtering, respectively. The computer saved the signals shown on the oscilloscope (Keysight DSOX 2014A, Santa Rosa, CA, USA). The minimum sampling frequency of the received signals was 1 MHz. The number of signals stacked for the high signal-to-noise ratio was 1024 [14].

For comparison, a fully saturated sand–silt mixture was prepared using the pluviation method, with a relative density *D_r_* = 70%. This specimen was dried in an oven. The degree of saturation for this specimen changes from *S* = 100% to 0%. The degree of saturation was calculated using the difference between the weight of the sample measured at the beginning of the experiment and the weight of the sample after the drying process. The shear waves were continuously measured as the degree of saturation decreased from 100% to 0%. The temperature inside the dryer was kept constant at 70 °C.

## 4. Experimental Results

This section reports the experimental results for shear wave velocity, as analyzed in the context of temperature and degree of saturation.

### 4.1. Shear Wave Signature

**Figure 5:**
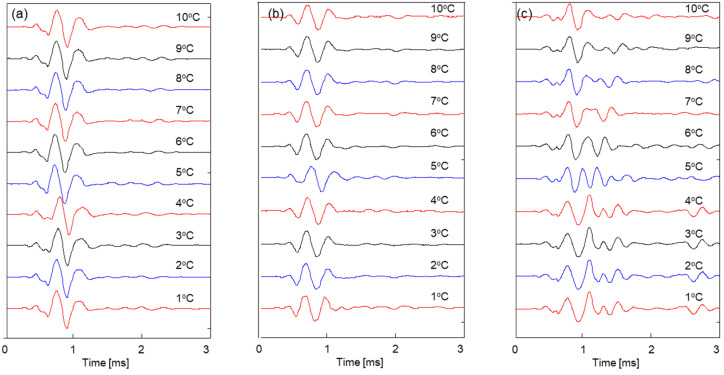
Shear wave signals for specimens with different degrees of saturation *S* during temperature changes from *T* = 10 to 1 °C (**a**) *S* = 2.5%; (**b**) *S* = 5%; and (**c**) *S* = 10%.

Figure 5 presents the shear wave signals for three specimens with degrees of saturation *S* = 2.5%, 5%, and 10% when they experience a temperature change from *T* = 10 °C to 1 °C. As the temperature decreases, the time to the first arrival of the shear wave tends to increase, indicating a decrease in shear wave velocity.

### 4.2. Shear Wave Velocity

**Figure 6:**
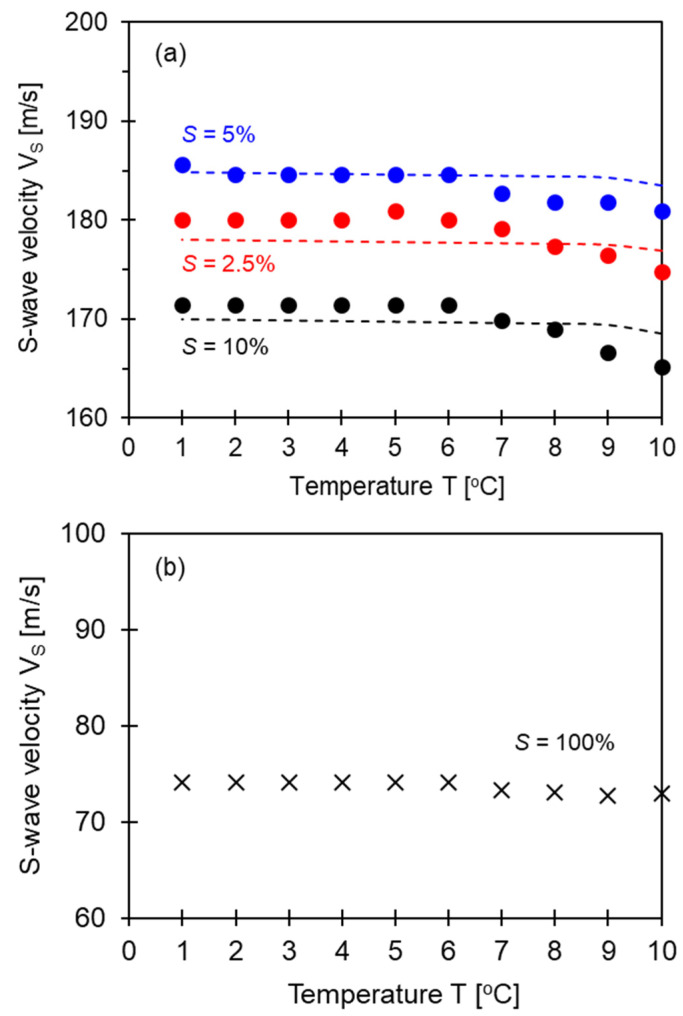
Shear wave velocity against temperature. Markers indicate the experimental results of the various water-saturated specimens during cooling, and dotted lines indicate the estimated shear wave velocity via the particle-scale analyses, considering the matric suction and temperature fluctuations. (**a**) Saturation (*S* = 2.5%, 5%, and 10%). (**b**) *S* = 100%.

The shear wave velocity is calculated using the following equation:

(2) $$V_{s}=\frac{L_{tip-tip}}{t_{s}}$$

where *L_tip−tip_* corresponds to the distance between the tips of the bender elements, and *t_s_* corresponds to the first arrival time of the shear wave signatures [2]. Figure 6 shows the shear wave velocity changes for the three specimens versus the temperature. At the beginning of the temperature drop (i.e., at *T* = 10 °C), the degree of saturation appears to be critical in determining the shear wave velocity. The shear wave velocity at *T* = 10 °C is *V_s_* = 175 m/s for *S* = 2.5%, *V_s_* = 181 m/s for *S* = 5%, and *V_s_* = 165 m/s for *S* = 10%. Overall, the degree of saturation *S* = 5% seems to be the best moisture condition for optimized matric suction to build up. It is widely known that matric suction is primarily affected by water saturation and tends to decrease with the increase in water saturation after reaching the optimized matric suction. Clearly, there is a transitional degree of saturation for shear wave velocity. However, there are pronounced increases in shear wave velocity with the temperature drop in all cases. Notably, the change in shear wave velocity for fully saturated (*S* = 100%, in Figure 6b) and dry specimens (*S* = 0%) was negligible during cooling, mainly because matric suction between particles was nearly zero. Details of water saturation effects on shear wave velocity are discussed in the next section. Geometry-based particle-scale analyses are also presented in the next section to enhance understanding of the temperature effect on shear wave velocity at a relatively low degree of saturation.

### 4.3. Shear Wave Velocity Change during Drying

**Figure 7:**
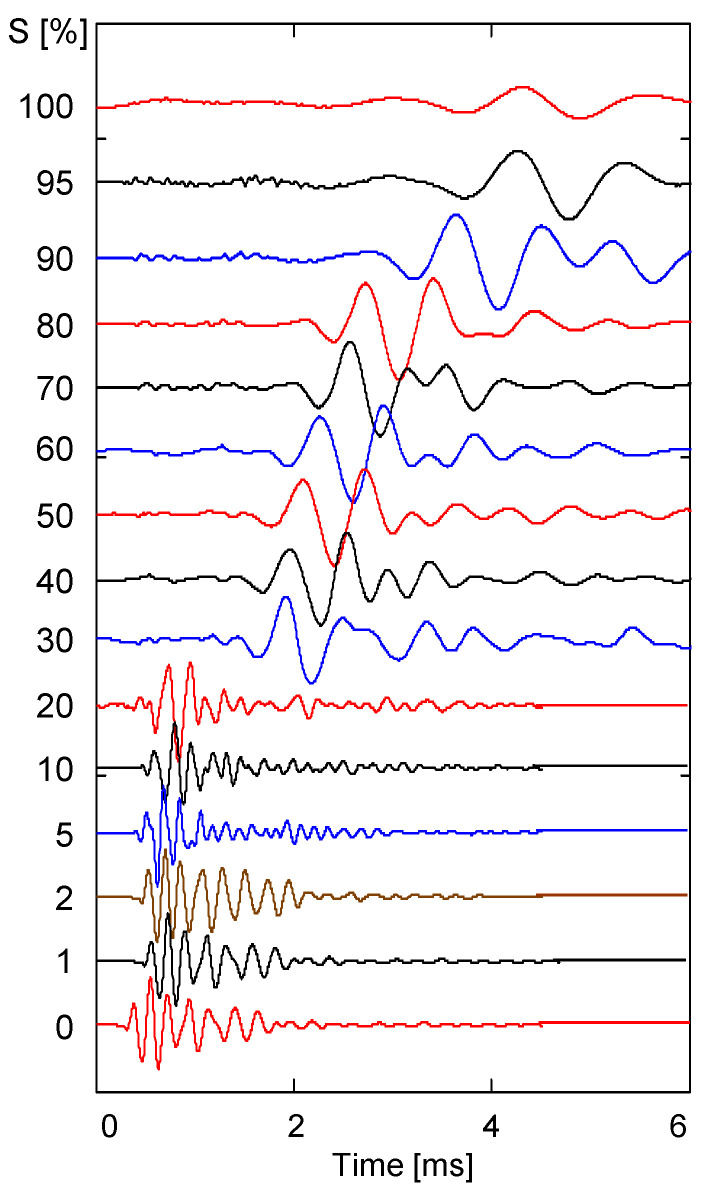
Shear wave signatures for the sand–silt mixture measured during the drying process.

After saturating the specimen mixed with sand and silt, shear wave signals were measured at a constant temperature (70 °C) during the drying process. Figure 7 shows the shear wave signal obtained when the degree of saturation changed from 100% to 0%. The results indicate that the shear wave signal change pattern was divided into three stages. The first step occurred in the saturation range of 100% to 90%, where the first arrival time of the shear wave rapidly decreased. The second stage occurred in the saturation range of 90% to 20%, where the first arrival time of the shear wave was relatively constant. Finally, the third step occurred in the saturation range of 20% to 0%, where the first arrival time of the shear wave rapidly decreased.

Similar to the shear wave signal, the resonant frequency change of the shear wave was divided into three stages. The resonance frequency was less than 1 kHz in the range of 100% to 90% saturation, about 2 kHz in the range of 90% to 20% saturation, and about 6 kHz in the range of 20% to 0% saturation. Figure 7 shows that there is a relationship between the change in the shear wave velocity and the change in the resonant frequency.

**Figure 8:**
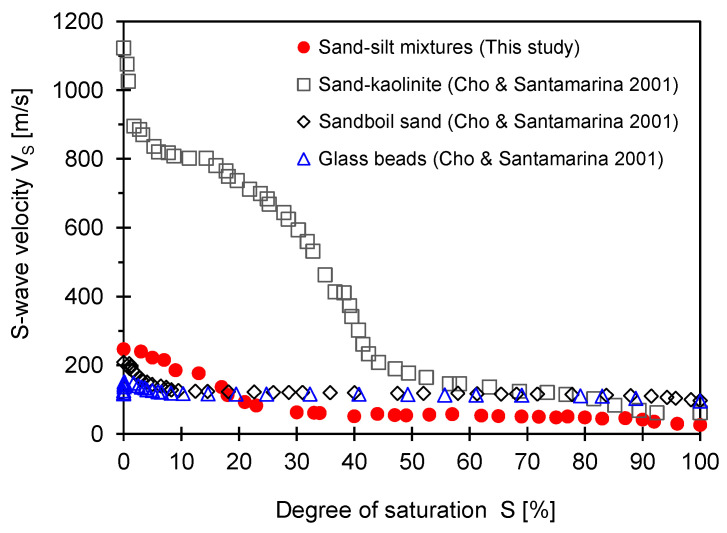
Shear wave velocity for the sand–silt mixture measured during the drying process.

The first arrival time was calculated from the shear wave signal shown in Figure 7, and the shear wave velocity was calculated using Equation (2). The shear wave velocity according to the degree of saturation is shown in Figure 8. The shear wave velocity and the degree of saturation are also divided into three stages. In the first stage, the shear wave velocity increased as the saturation decreased from 100% to 90% saturation. The second stage represents the behavior of unsaturated soil with a saturation range of 90% to 20%, and the shear wave speed increases when the graph shows a gentle slope. In the third stage, the shear wave velocity increased rapidly in the saturation range of 20% to 0% and showed the largest speed change.

For comparison, data extracted from previous studies overlapped with the results obtained in this paper. The results indicate that shear wave velocity for the sand–silt mixture follows a similar trend compared to other sand–fine mixtures. Furthermore, there was a slight increase in shear wave velocity at a low degree of saturation (i.e., *S* ~ 20 to 0%) in all cases except for the sand–kaolinite mixture. There seems to be a cementation effect at the degree of saturation *S* = 0%; otherwise, shear wave velocity should decrease at *S* = 0%. The shear wave velocity at *S* = 20% was *V_s_* = 116 m/s, while the shear wave velocity at *S* = 10% was 186 m/s. Hence, the shear wave velocity depends on the degree of saturation. The effect of temperature on shear wave velocity could be more significant at a low degree of saturation rather than at a high degree of saturation.

## 5. Analyses and Discussion—Temperature-Dependent Shear Wave Velocity

The void ratio for soils e changes with overburden pressure and decreases as the vertical effective stress *σ*′ increases. An asymptotically correct soil compaction model is therefore used to capture changes in the void ratio with effective stress. This compaction model includes two extreme constitutive parameters: the void ratio *e_L_* at extremely low effective stress (i.e., *σ*′ is close to zero) and the void ratio *e_H_* at extremely high effective stress (i.e., *σ*′ is close to infinite) [15,16]:

(3) $$e_{z}=e_{H}+\left(e_{L}-e_{H}\right)\exp\left[-{\left(\frac{\sigma \prime }{\sigma_{c}^{\prime }}\right)}^{\eta }\right]$$

where the model parameter *η* reflects the sensitivity of the void ratio to effective stress, and *σ*′*_c_* is the characteristic effective stress (note: *η* = 1/3 in this paper). Once again, the void ratio of soils changes with effective stress, yet the void ratio follows asymptotic trends at very low and high effective stresses. Adopting reference sediment No. 5 and associated model parameters [15], the void ratio decreases with an increase in effective stress (note: input model parameters for Equation (3) include *e_L_* = 0.91, *e_H_* = 0.2, and *σ_c_* = 2000 kPa). As the initial void ratio *e_o_* corresponding to *D_r_* = 70% is known, the void ratio can be anticipated at a given effective stress. Then, this paper estimated the capillary pressure assuming a plate-like particle shape with a thickness t and a pore diameter *d_p_* (note: it could be reasonable to assume a plate-like particle shape for a sand–silt mixture). This geometrical assumption enables the definition of the specific surface *S_s_*, which is the ratio between the total surface area *A_T_* and its mass *M*:

(4) $$S_{s}=\frac{A_{T}}{M}=\frac{2}{t\rho_{m}}$$

Based on the definition, the void ratio e is the ratio between the volume of the void *V_v_* and the volume of solids *V_s_*:

(5) $$e=\frac{V_{v}}{V_{s}}=\frac{d_{p}}{t}$$

Then, geometric analyses lead to the definition of pore diameter *d_p_* in terms of void ratio *e*, specific surface *S_s_*_,_ and mineral density *ρ_m_*:

(6) $$d_{p}=e\cdot t=\frac{2e}{S_{s}\rho_{m}}$$

For unsaturated soils, the capillary pressure ∆*u* (or matric suction) increases, and this is a function of interfacial tension *T_s_* and pore radius *R* (or pore diameter *d_p_*). Combining Equations (4)–(6), the capillary pressure ∆*u* can be expressed as follows [6]:

(7) $$\Delta u=\frac{2T_{s}}{R}=\frac{4T_{s}}{d_{p}}=2\frac{T_{s}S_{s}\rho_{m}}{e}$$

As addressed above, the interfacial tension decreases with temperature [9]:

(8) $$T_{s}=76.05-0.148T-1.619\times 10^{-4}T^{2}$$

where *T* is the temperature (°C). Then, the capillary pressure increases as the temperature decreases. The temperature range of the obtained relationship in Equation (8) was between 5 °C and 45 °C [9], and this paper assumes that this relationship holds up to 1 °C.

The shear modulus for soil skeleton *G_sk_* at the small-strain level can be expressed as a function of the shear modulus of the mineral that makes the grain *G_m_*, Poisson’s ratio *ν* of the mineral, and stresses in the direction of principal soil fabric *σ_p_* and *σ_m_* [17]:

(9) $$G_{sk}=\frac{9G_{m}{}^{\frac{2}{3}}}{6-5\upsilon_{m}}{\left(\frac{1-\upsilon_{m}}{6}\right)}^{\frac{1}{3}}{\left(\frac{\sigma_{p}+2\sigma_{m}}{3}\right)}^{\frac{1}{3}}$$

For the first-order approximation, shear wave velocity is mainly determined via the shear modulus for soil skeleton *G_sk_* and the density of the sand–silt mixture. The shear wave velocity *V_s_* is

(10) $$V_{S}=\sqrt{\frac{G_{sk}}{\rho_{mix}}}$$

The dotted lines overlapped on the data in Figure 6 indicate the particle-scale analyses that predict the change in shear wave velocity for unsaturated soils subjected to cooling. These geometry-based models combine soil index properties, anticipated pore diameter, capillary pressure, and temperature-dependent interfacial tension (note: model parameters used for model prediction involve initial void ratio *e_o_* = 0.69, specific surface *S_s_* = 0.2 m^2^/g; mineral density *ρ_m_* = 2650 kg/m^3^, *G_m_* = 1.45 × 10^9^ Pa, *ν* = 0.31, *ρ_mix_* = 1917 kg/m^3^). Once again, the void ratio is updated as the interfacial tension increases with each temperature drop, followed by an increase in capillary pressure and an increase in effective stress. These simple analyses were conducted based on a single-pore scenario. However, soils consist of a wide range of grain sizes; therefore, multi-pore scenarios prevail in nature. For further data interpretation, multi-pore analyses should be conducted to accurately predict shear wave velocity under unsaturated conditions when subjected to cooling.

Once again, the capillary-driven matric suction caused by the difference between air pressure and water pressure is an important factor that significantly affects the behavior of unsaturated soils. Various studies have been conducted to provide quantitative solutions. After simplifying the arrangement of particles (simple cubic packing, tetrahedral packing, and packing with binary-sized particles), many researchers have tried to theoretically obtain the matric suction as a function of the degree of saturation using the geometric relationship between the particles and the meniscus [5,18,19,20,21]. Because the interfacial tension constituting the capillary absorption capacity varies with temperature, the soil–water characteristic curve (SWCC) is expressed as a function of temperature. Many investigations have compared the theoretical and experimental values of the theoretically predicted capillary-driven matric suction [22,23,24] and have directly obtained capillary adsorption capacity according to temperature change experimentally [25,26,27]. Comparison between theoretically predicted computation and experimental results reveals that the experimentally measured matric suction is much larger than the theoretically predicted value, and the matric suction according to the temperature change is also larger in the experimental value than the theoretical value.

While the temperature of the sample changes from 10 °C to 1 °C, the increase in interfacial tension is about 2%. The increase in effective stress due to surface tension is also about 2%. Since the typical exponent for shear wave velocity in the power function of effective stress has a value in the range of 0.15 to 0.25, the effect of interfacial tension due to temperature change can be predicted. The expected increase in shear wave velocity is less than 2% compared to the initial shear wave velocity. The increase in shear wave velocity obtained through the experiments varies from 10% to 35%, depending on the degree of saturation as the temperature changes (Figure 8). The reason the increase in measured shear wave velocity is greater than the increase in shear wave velocity expected from the quantitative increase in interfacial tension due to temperature change is that the increase in shear wave velocity is not only due to interfacial tension but also the temperature-induced water volume change, the change in air volume due to the temperature change, the effect of solutes dissolved in water, and the temperature sensitivity of the contact angle [28,29,30].

Figure 6 and Figure 8 show that the shear wave velocity change pattern is roughly divided into three stages, according to the degree of saturation. The first stage occurs in a saturation range of 100% to 90%, with a slight increase in shear wave velocity; the second occurs in a saturation range of 90% to 20%, with a moderate increase in shear wave velocity; and the third stage occurs in a saturation range of 20% to 0%. In the first and second stages, the shear wave velocity trends for specimens prepared via the tamping method at each saturation level and the dried sample are similar; however, in the third stage (i.e., the saturation range of 20% to 0%), the shear wave velocity trends are completely different from each other. This difference may result from the increase in the number of contacts caused by the movement of particles that occurs when the fully saturated sample is dried and the change in stiffness due to the effect of residual compressive stress. In addition, the number of pores and the uneven distribution of particles can occur in specimens where the degree of saturation is controlled via the tamping method. Those factors are also considered to influence the different patterns of the change in shear wave velocity seen in specimens prepared via the air pluviation composition method. Figure 5 and Figure 7 show that the frequency of the shear wave signatures appears differently at the same saturation depending on the sample preparation method. The resonance frequency of the 10% saturation specimen prepared via the tamping method is smaller than that of the 10% saturation specimen in the drying process. The different resonance frequencies may be due to the change in stiffness caused by the effect of the residual compressive stress and the increase in the number of contacts between particles.

## 6. Conclusions

This research investigated the changes in shear wave velocity for unsaturated and fully saturated specimens subjected to a temperature change from 10 °C to 1 °C. This experimental research outlined the design of a nylon cell equipped with bender elements in a cross-hole configuration to measure the shear wave velocity for nine sand–silt mixtures with different degrees of saturation (*S* = 0%, 2.5%, 5%, 10%, and 100%). Salient conclusions are presented below.

As the temperature decreases from 10 to 1 °C, the shear wave velocity for specimens with a low degree of saturation increases. Particle-scale analyses that predict the change in shear wave velocity for unsaturated soils subjected to cooling overlap with experimental results (i.e., *V_s_* versus temperature). These geometry-based models combine soil index properties, anticipated pore diameter, capillary pressure, and temperature-dependent interfacial tension.

The void ratio is updated as the interfacial tension increases with each temperature drop, followed by an increase in capillary pressure and an increase in effective stress. These simple analyses are conducted based on a single-pore scenario. However, soils consist of a wide range of grain sizes; therefore, multi-pore scenarios prevail in nature. For further data interpretation, multi-pore analyses should be conducted to accurately predict shear wave velocity under unsaturated conditions subjected to cooling.

When calculating the shear modulus of unsaturated soil on the micro-deformation scale using shear wave velocity, the shear wave velocity is affected by temperature for various reasons. Therefore, the role of temperature-dependent interfacial tension should be considered in determining geosystem design parameters. Further research is recommended on the simultaneous measurement of capillary force and shear wave velocity under various saturation and temperature conditions.

## Figures and Tables

**Table 1:** Soil index properties.

Property	Sand	Sand–Silt Mixture
Specific gravity *G_s_*	2.62	2.57
Mean grain size *D*_50_	0.45	-
*e_max_*	0.82	0.74
*e_min_*	0.56	0.47

## Data Availability

The data presented in this study are available upon reasonable request from the corresponding author.
